# Does Sleep Mediate the Association between School Pressure, Physical Activity, Screen Time, and Psychological Symptoms in Early Adolescents? A 12-Country Study

**DOI:** 10.3390/ijerph16061072

**Published:** 2019-03-25

**Authors:** Ann Vandendriessche, Ariane Ghekiere, Jelle Van Cauwenberg, Bart De Clercq, Karlien Dhondt, Ann DeSmet, Jorma Tynjälä, Maïté Verloigne, Benedicte Deforche

**Affiliations:** 1 Department of Public Health, Faculty of Medicine and Health Sciences, Ghent University, Corneel Heymanslaan 10, 4K3, B-9000 Ghent, Belgium; ariane.ghekiere@zorg-en-gezondheid.be (A.G.); jelle.vancauwenberg@ugent.be (J.V.C.); b.declercq@ugent.be (B.D.C.); benedicte.deforche@ugent.be (B.D.); 2 Fund for Scientific Research Flanders (FWO), Egmontstraat 5, B-1000 Brussels, Belgium; 3 Department of Psychiatry: Pedicatric Sleep Center, Ghent University Hospital, Corneel Heymanslaan 10, B-9000 Ghent, Belgium; karlien.dhondt@ugent.be; 4 Department of Movement and Sports Sciences, Faculty of Medicine and Health Sciences, Ghent University, Watersportlaan 2, B-9000 Ghent, Belgium; ann.desmet@ugent.be (A.D.); maite.verloigne@ugent.be (M.V.); 5 Faculty of Sport and Health Sciences, PO Box 35, FI-40014 University of Jyväskylä, Finland; jorma.a.tynjala@jyu.fi; 6 Physical Activity, Nutrition and Health Research Unit, Faculty of Physical Education and Physical Therapy, Vrije Universiteit Brussel, Pleinlaan 2, B-1050 Brussels, Belgium

**Keywords:** mental health, adolescents, sleep

## Abstract

This study examines the mediating role of sleep duration and sleep onset difficulties in the association of school pressure, physical activity, and screen time with psychological symptoms in early adolescents. Data were retrieved from 49,403 children (13.7 ± 1.6 years old, 48.1% boys) from 12 countries participating in the World Health Organization (WHO) “Health Behaviour in School-aged Children” 2013/2014 study. A validated self-report questionnaire assessed psychological symptoms (feeling low, irritability or bad temper, feeling nervous), school pressure, physical activity (number of days/week 60 min moderate-to-vigorous), screen time, sleep duration on week- and weekend days, and perceived difficulties in getting asleep. Multilevel mediation analyses were conducted. School pressure and screen time were positively associated with psychological symptoms, whereas physical activity was negatively associated. With the exception of sleep duration in the association between physical activity and psychological symptoms, all associations were significantly mediated by sleep duration on week- and weekend days and sleep onset difficulties. Percentages mediated ranged from 0.66% to 34.13%. This study partly explains how school pressure, physical activity, and screen time are related to adolescents’ psychological symptoms. Future interventions improving adolescents’ mental well-being could target schoolwork, physical activity, and screen time, as these behaviours are directly and indirectly (through sleep) related to psychological symptoms.

## 1. Introduction

The prevalence of mental health problems among adolescents (i.e., 12–18-year-olds) is alarming: an average of 20% of European adolescents suffer from developmental, behavioral, or emotional problems, and about 12.5% have a clinically diagnosed mental disorder [1,2]. The prevalence of major depression among adolescents in Europe ranges between 7.1% and 19.4% [3]. Furthermore, poor mental health in adolescence tracks into adulthood [4]. Given the high prevalence of mental health problems in European adolescents, the prevention of these problems is an important public health issue. 

Various factors have been shown to be associated with mental health conditions in adolescents. For example, a positive association was demonstrated between perceived school pressure and mental health problems in adolescents [5,6,7]. Higher exposure to screen time, Internet, and social media has also been related to more psychological symptoms among adolescents, such as lower life satisfaction and self-image, and higher scores on depression and anxiety scales [8,9]. On the other hand, physical activity has been identified as a protective factor against mental health problems [5]. Regular physical activity has shown to lower the risk of depression and anxiety, and to be associated with higher self-esteem [8,10].

Although the direct associations of school pressure, physical activity and screen time with mental health have been investigated in several studies, there is little research investigating the underlying mechanisms of these associations. Sleep duration and sleep onset difficulties (which is an indicator of sleep quality) could play an important mediating role, as school pressure, physical activity and screen time are related with sleep quantity and quality [11,12,13,14,15]. To our knowledge, only two studies have examined sleep duration or sleep onset difficulties as a possible mediating factor between screen time and mental health outcomes. A first study in Italian 15-year-olds [16] found that sleep onset difficulties partly mediates the association between computer use and psychological symptoms. A second study in 15-year-olds from Finland, France, and Denmark found that sleep duration partially mediates the association between computer use and health symptoms [17]. However, only computer use was investigated as specific screen-based behavior in those studies. As higher levels of television watching and smartphone use were also shown to be associated with less sleep and more sleep onset difficulties [11,13,18], it is relevant to investigate sleep quantity and quality as a possible mediating factor. In addition, to our knowledge, the mediating role of sleep duration or difficulties has not yet been examined in the association of school pressure and physical activity with psychological symptoms. Given the share of mental disorders taking onset at a young age [19], it is important to explore these associations among young adolescents prior to the onset of mental health problems. The purpose of this paper was to investigate the mediating role of sleep duration and sleep onset difficulties in the association of screen time, school pressure, and physical activity with psychological symptoms in 11- to 15-year-old adolescents from 12 European countries. 

## 2. Materials and Methods

### 2.1. Sample and Protocol

Data from 12 countries (i.e., Belgium (French-speaking), Switzerland, Germany, Estonia, Spain, Finland, Hungary, Latvia, Scotland, Sweden, Slovenia, and Wales) within the “Health Behaviour in School-aged Children: WHO Collaborative Cross-National survey/study (HBSC) 2013–2014” were used. The Health Behaviour in School-Aged Children (HBSC) study is a World Health Organisation (WHO) collaborative, cross-national study conducted among adolescents aged 11 to 15 years from more than 40 countries and regions across Europe and North America. Every four years, data are collected on young people’s well-being, health behaviours, and life circumstances using a structured questionnaire. Every participating country can choose to add an optional package of questions to this questionnaire. To ensure comparability of the data, each country performs a translation-back translation on the country-specific questionnaire [20]. Every country collects data according to a standardized international protocol, which can be downloaded from http://www.hbsc.org/methods/index.html. Ethical approval is obtained at the national or regional level. Adolescents complete the international standard questionnaire developed by the research network of HBSC in the classroom after instruction by a teacher or a trained interviewer. Data collection happened between October 2013 and December 2014, during a different time of year for each included country. More details of the study protocol can be found elsewhere [21].

### 2.2. Measures

For the purpose of this study, the following variables were used: socio-demographic variables (age, gender and socio-economic status), school pressure, physical activity, screen time and sleep duration. Furthermore, psychological symptoms were used as a proxy for mental health, i.e., feeling low, irritability or bad temper, and feeling nervous. Furthermore, sleep onset difficulties (as a proxy for sleep quality) were assessed. According to Buysse et al., sleep quality is composed out of seven components, one of them being sleep onset difficulties [22]. Table 1 provides an overview of each variable. As sleep duration was part of an optional package within the HBSC-study, only countries that had questioned all above-mentioned variables were included in this study. 

### 2.3. Statistical Analysis

Statistical analyses were conducted in RStudio version 3.3.2 with statistical significance set at a *p*-value of 0.05. All analyses were adjusted for age, gender, and socio-economic status. Adolescents were clustered within classes, schools, and countries. For analyses in the present study, only two levels were included in the multilevel structure. Only the clustering of individuals within classes was taken into account, as it had the largest explained variance of the three levels (7% for class-level versus 0.8% for school- and country-level). To assess the overall relationship of school pressure, physical activity, and screen time with psychological symptoms, multilevel linear regression analyses were used. To assess the mediating role of sleep duration on week and weekend days and sleep onset difficulties on the associations of school pressure, physical activity, and screen time with psychological symptoms, multilevel mediation analysis was performed using the lme4 and mediation package. The mediation package requires all cases to have complete data, so participants with missing values on any of the involved variables were withheld from the analyses. This resulted in an analytic sample of 49,403 participants for all analyses. Out of these, 9433 participants were excluded due to missing data.

**Figure 1:**
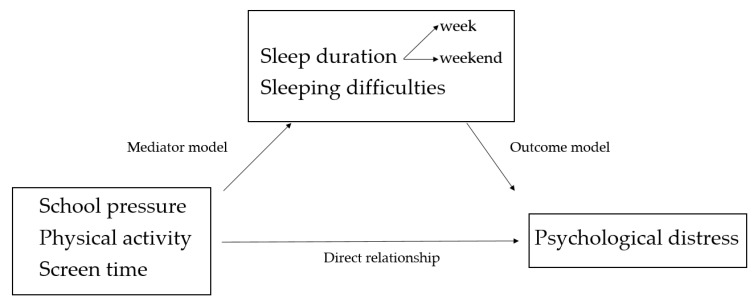
Visual representation of the model tested. It assesses the direct relationship of school pressure, physical activity, and screen time with psychological symptoms and the mediation of this relationship by sleep duration and sleep onset difficulties.

Three consecutive steps were taken [30,31]. First, a multilevel regression equation was created to assess the association between school pressure, physical activity, and screen time, with (a) sleep duration on weekdays, (b) sleep duration on weekend days, and (c) sleep onset difficulties (the mediator models). Sleep duration on week- and weekend days and sleep onset difficulties were mutually used as covariates (i.e. in the equation for sleep duration on weekdays, sleep duration on weekend days, and sleep onset difficulties were included as covariates). Secondly, the association between (a) sleep duration on weekdays, (b) sleep duration on weekend days, and (c) sleep onset difficulties with psychological symptoms was tested (the outcome models). Covariates used here were school pressure, physical activity and screen time. Finally, the mediating role of (a) sleep duration on weekdays, (b) sleep duration on weekend days, and (c) sleep onset difficulties on the association between school pressure, physical activity, and screen time with psychological symptoms was assessed. The visual representation of the mediation analyses is shown in Figure 1.

## 3. Results

### 3.1. Descriptive Characteristics of the Analytic Sample

The mean age of the study sample (48.1% boys) was 13.74 (±1.63) years (*n* = 49.403). Most participants (62.9%) were not or a little pressured by schoolwork, 24.2% felt some pressure by school work, and 12.9% were pressured a lot. Participants reported to be physically active for an average of 4.18 days (±1.99) per week for at least 60 min per day. Mean screen time was 6 h and 33 min per day (±3 h and 49 min). The mean reported score for psychological symptoms was 3.75 (±1.07) on a five-point scale, with 0 as never experiencing psychological symptoms and 5 as experiencing them every day. Mean sleep duration was 8 h and 19 min (±1 h and 10 min) on weekdays and 9 h and 49 min (±1 h and 35 min) on weekend days. A mean of 2.22 (±1.40) on a five-point scale was observed for experiencing difficulties in getting to sleep. 

### 3.2. Direct Relationship of School Pressure, Physical Activity, and Screen Time with Psychological Symptoms

Direct relationships between school pressure, physical activity, screen time and mental well-being were all significant (*p* < 0.001). Experiencing some or a lot of school pressure (in comparison to none or a little school pressure) was associated with an increase in psychological symptoms of 0.40 (95% CI = −0.42; −0.38) and 0.86 (95% CI = −0.89; −0.83) on a five-point scale, respectively. An extra day of physical activity was associated with a decrease in psychological symptoms of 0.02 on a 5-point scale (95% CI = 0.02; 0.03). One extra hour of screen time per day was associated with an increase in psychological symptoms of 0.04 on a 5-point scale (95% CI = −0.04; −0.04).

### 3.3. Mediation Analysis

Results of the mediation analysis are shown in Table 2. All associations were significant (*p* < 0.001). The mediator model, the outcome model, and percentages mediated are discussed below for each potential mediator.

#### 3.3.1. Sleep Duration on Weekdays

Experiencing some or a lot of school pressure (in comparison to none or a little school pressure) was associated with a decrease in sleep duration of 5.5 min per day (*β* = −0.09, 95% CI = −0.11; −0.07) and 15 min per day (*β* = −0.25, 95% CI = −0.28; −0.22) on weekdays, respectively. Sleep duration on weekdays also decreased by 0.6 min (*β* = −0.01, 95% CI= −0.01; −0.01) with each extra day of physical activity a week, and by 4.2 min (*β* = −0.07, 95% CI = −0.07; −0.07) with each extra hour of screen time a day. 

A higher sleep duration on weekdays was associated with lower psychological symptoms (*β* = −0.13, 95% CI = −0.13; −0.12). Sleep duration on weekdays accounted for 2.85% (95% CI = 2.21; 3.56) of the relationship between some school pressure and psychological symptoms, while it accounted for 3.62% (95% CI = 3.15; 4.08) of the relationship between a lot of school pressure and psychological symptoms. Sleep duration accounted for 22.16% (95% CI = 20.29; 24.30) of the relationship between screen time and psychological symptoms. Physical activity was positively associated with psychological symptoms through sleep duration, whereas the direct relationship indicated an inverse relationship between physical activity and psychological symptoms (see Table 2). Therefore, this can be considered as an inconsistent mediation.

#### 3.3.2. Sleep Duration on Weekend Days

Experiencing some or a lot of school pressure (in comparison to none or a little school pressure) was associated with a decrease of sleep duration of 4.56 min per day (*β* = −0.08, 95%CI = −0.11; −0.04) and 11.82 min per day (*β* = −0,20, 95% CI = −0.24; −0.15) on weekends, respectively. Sleep duration on weekend days also decreased by 1.8 min (*β* = −0.03, 95% CI = −0.04; −0.02) with each extra day of physical activity a week, and by 3 min (*β* = −0.05, 95% CI = −0.05; −0.05) with each extra hour of screen time a day. 

A higher sleep duration on weekends was associated with lower psychological symptoms (*β* = −0.04, 95% CI= −0.03; −0.04). Sleep duration on weekends accounted for 0.66% (95% CI = 0.37; 1.02) and 0.79% (95% CI = 0.60; 1.03) of the relationship between school pressure (some and a lot) and psychological symptoms, whereas it accounted for 4.49% (95% CI = 3.73; 5.37) of the relationship between screen time and psychological symptoms. The mediation of sleep duration on weekends on the association between physical activity and psychological symptoms was also inconsistent (cf. mediation of sleep duration on weekdays on the association between physical activity and psychological symptoms).

#### 3.3.3. Difficulties in Getting to Sleep

Experiencing some or a lot of school pressure (in comparison to none or a little school pressure) was associated with an increase of sleep onset difficulties of 0.32 (*β* = 0.32, 95% CI = 0.30; 0.35) and 0.73 (*β* = 0.73, 95% CI = 0.69; 0.77) on a five-point scale, respectively. More physical activity was associated with lower levels of sleep onset difficulties: sleep onset difficulties decreased by 0.03 on a five-point scale with each extra day of physical activity a week (*β* =−0.03, 95% CI =−0.03; −0.02). Finally, sleep onset difficulties increased by 0.04 with each extra hour of screen time a day (*β* = 0.04, 95% CI = 0.04; 0.05). 

Higher levels of sleep onset difficulties were associated with higher levels of psychological symptoms (*β* = 0.30, 95% CI = 0.30; 0.31). Sleep onset difficulties accounted for 24.60% (95% CI = 22.28; 26.58) and 25.80% (95% CI = 24.60; 27.20), respectively, of the relationship between school pressure (some and a lot) and psychological symptoms. Additionally, sleep onset difficulties accounted for 33.56% (95% CI = 26.36; 41.61) of the total relationship between physical activity and psychological symptoms, and for 34.13% (95% CI = 31.74; 36.63) of the total relationship between screen time and psychological symptoms.

## 4. Discussion

This study showed that more school pressure, fewer days of engaging in sufficient physical activity, and higher levels of screen time were associated with more psychological symptoms in European adolescents. Next to the direct associations, sleep duration on week- and weekend days and sleep onset difficulties were shown to be mediators in all associations, except for sleep duration in the association between physical activity and psychological symptoms. 

The mediating role of sleep in the associations first implies that the sleep-related variables were related to psychological symptoms: per increasing hour of sleep on week- or weekend days, the score for psychological symptoms decreased by 0.13 and 0.04 on a 5-point scale, respectively. Further, per one unit increase on a five-point scale for experiencing sleep onset difficulties, psychological symptoms increased by 0.30 on a five-point scale. Although our findings are cross-sectional, they suggest that it is important for adolescents’ well-being to focus on good sleep quantity and quality. Moreover, our results are in line with a previous study, in which better sleep quantity and quality were related to a better mental health status in adolescents [32,33]. The magnitude of the associations was somewhat higher for sleep onset difficulties than for sleep duration in our study. Studies investigating the relationship between sleep and other outcomes have found similar results: for example, sleep quality was found to have a stronger association with school performance [34] and mood [35] than sleep duration in adolescents. 

Regarding the direct associations, adolescents’ feeling of school pressure and levels of physical activity and screen time were all significantly associated with adolescents’ psychological symptoms. Experiencing some or a lot of school pressure compared to none or a little was associated with a decrease in psychological symptoms by 0.4 and 0.9 on a five-point scale, respectively. In a previous Norwegian study, school stress was positively related to adolescents’ psychological symptoms as well [36], which is now confirmed on a broader European level. Furthermore, this relationship between school pressure and psychological symptoms was mediated for about a fourth (some: 24.6%; a lot: 25.8%) by sleep onset difficulties. A previous study of Thomsen et al. [37] also showed that ruminating (i.e., about school work) was associated with a lower sleep quality and depressive mood. In our study, the mediating role of sleep quantity in the relationship between school pressure and mental health was quite modest (all smaller than 3.6%), although adolescents experiencing a lot of school pressure slept 15 min less on weekdays and almost 12 min less on weekend days, compared to adolescents perceiving no or a little school pressure. Nevertheless, these results suggest an important role for schools to manage the pressure caused by school duties, as perceived school pressure in adolescents is associated with both unhealthy sleep as well as psychological symptoms. 

Furthermore, being physically active was inversely associated with adolescents’ psychological symptoms. However, the magnitude of the association was rather small: for each extra day per week of being physically active for at least 60 min, the score on psychological symptoms decreased only by 0.02 on a five-point scale. This is in line with the results of a previous review, which showed some evidence for a negative association between physical activity and levels of depression and anxiety in adolescents [8]. The mediation analyses further showed that about 33% of the relationship between physical activity and psychological symptoms could be explained by decreased sleep onset difficulties. However, in view of the weak relationship between psychological symptoms and physical activity, this mediation might be less important to elaborate on. In addition, the mediating role of sleep quantity was inconsistent, as higher levels of physical activity were associated with less sleep duration on week- and weekend days. This could suggest that time spent in physical activities might be at the cost of sleep duration (e.g., late-night football practice on weekdays, or getting up early on weekend days to play sports). In other words, physical activity performed during the day may enhance sleep quantity and quality, whereas physical activity performed in the evening could impair sleep quantity and quality by prolonging the sleep onset [38], although this has been questioned by other studies [39]. To disentangle this further, it might be interesting to include questions about the time of day during which physical activity was performed in future research. 

Finally, screen time was positively related to psychological symptoms: per extra hour of screen time per day, adolescents’ psychological symptoms increased with 0.04 on a 5-point scale. This is in line with findings of a meta-analysis by Asare, in which the association of adolescents’ screen time with mental health was found to be negative, but rather small [40]. Another study also found that screen time in adolescence was inversely associated with mental wellbeing in adulthood [41]. Our findings indicate that limiting screen time in adolescence could be important for their mental health, although it has to be kept in mind that our analyses were cross-sectional. Further, the three sleep-related variables were all significant mediators in this association, although the percentage mediated by sleep duration on weekend days was rather limited (4.5%). The difference with the percentage mediated by sleep duration on weekdays (22.2%) could perhaps be due to differences in the timing of screen time. As adolescents are at school during the day on weekdays, most screen time probably occurs in the evening, whereas screen time on weekend days could be more spread throughout the entire day; however, this is just a hypothesis, and requires further investigation. Finally, as sleep onset difficulties were also a significant mediator in the association between screen time and psychological symptoms, this implied a positive association between sleep quality and screen time as well. Indeed, it has been found that the blue light emitted by screens causes difficulties in getting to sleep [11,42].

Although our study has a cross-sectional design, our results might guide the development of future interventions focusing on improving adolescents’ mental well-being. These interventions could focus on lowering (perceptions of) school pressure, promoting physical activity, and limiting screen time. Focusing on these three factors might not only be important because of their direct association with mental health, but also because of their indirect association via sleep quantity and quality. Although the direct association has been suggested by earlier research [43], this indirect mechanism was not yet demonstrated, which is an important contribution to the literature. This study suggests that when interventions focus on the three above-mentioned behaviours to improve mental health in adolescents, sleep might be enhanced as well, which has a number of other positive effects on physiologic, neuro-cognitive, and emotional functions. In addition, directly focusing on sleep hygiene would be a valuable addition to mental health promotion programs as well, as adequate sleep is also associated with higher levels of physical activity, which is associated with better mental health, and with reduced stress levels [44]. To our knowledge, strategies focusing on these factors were not yet combined in an intervention to improve mental health in adolescents. Current intervention studies often focus on mental illness prevention rather than mental health promotion. An overview of interventions by Das et al. showed that cognitive behavioural therapy was commonly used to improve mental health in adolescents. A few interventions included physical activity programmes (i.e. walking to school, outdoor adventure, sports, and physical fitness programmes), which had small effects on anxiety and depression but rather large effects on self-esteem. One study focused on media literacy in order to promote a healthy self-image, but no intervention effects were found [45].

An important strength of this study is the large sample of adolescents (*n* = 49,403) from 12 European countries. There are, however, several limitations that need to be acknowledged as well. As discussed before, due to the cross-sectional design of this research, causal associations are potentially reversed or bi-directional. Indeed, adolescents’ psychological symptoms could also affect the degree of perceived school pressure, physical activity, and screen time. Longitudinal or experimental research is needed to confirm our findings. Secondly, although all associations between the independent variables and sleep duration were found to be significant, the magnitude of the associations was rather small. It is possible that statistical significance was reached for such small associations because of the large sample size. In addition, although statistically significant, there is no clinical relevance of, i.e., 4.56 min of extra sleep. Furthermore, there are no cut-off points or treshold for the scale of psychological symptoms, which makes the estimation of the clinical significance of the results (i.e. decrease of psychological symptoms of 0.13 and 0.04 on a five-point scale) difficult. To avoid this, future research could use the Depression Anxiety Stress Scale (DASS) [46], as this scale does provide cut-off scores. Finally, this research was limited by the measurements used in the self-reported HBSC-study. The HBSC study collects data on a variety of themes; each theme is therefore questioned as briefly as possible. Some variables (such as school pressure or sleep onset difficulties) were therefore constrainedly based on a single item. Psychological symptoms were used as a proxy for mental health. A more comprehensive measurement of mental health, such as the KIDSCREEN-10 [47], would benefit future research. Sleep onset difficulties were used as a proxy for sleep quality, while sleep quality is defined by several components. Ideally, all seven components of sleep quality (sleep quality, sleep latency, sleep duration, habitual sleep efficiency, sleep disturbances, sleeping medication use, and daytime dysfunction), according to Buysse et al., should be measured [22].

## 5. Conclusions

This study showed that more school pressure, fewer days of engaging in sufficient physical activity, and higher levels of screen time were associated with more psychological symptoms in adolescents from 12 European countries. Next to the direct associations, sleep duration on week- and weekend days and sleep onset difficulties were shown to be mediators in all associations, except for sleep duration in the association between physical activity and psychological symptoms. Future interventions improving adolescents’ mental wellbeing might therefore target adolescents’ perceptions of school pressure, physical activity, and screen time, as these factors are directly and indirectly (through sleep) related to psychological symptoms.

## Figures and Tables

**Table 1:** Overview of used variables.

Variable	Question	Response Categories	Data Processing	Reference
Socio-economic status (SES)	Family Affluence Scale III consisting of six items: number of cars family owns, having own bedroom, number of computers family owns, number of bathrooms at home, having a dishwasher at home, and number of travels for a vacation last year.	Number of cars family owns: None (0), one (1), two or more (2) Own bedroom: No (0), yes (1) Number of computers: None (0), one (1), two (2), more than two (3) Number of bathrooms: None (0), one (1), two (2), more than two (3) Dishwasher: No (0), yes (1) Family holidays: Not at all (0), once (1), twice (2), more than twice (3)	A sum score was calculated (range 0–9) and transformed into a continuous variable by performing a ridit transformation (range 0–1). In that way, the Family Affluence Scale indicates the relative material position within society (i.e. country). The scale also takes population heterogeneity for age and gender into account. Higher values reflect higher SES.	Material affluence was used as a measure of SES [23], using the Family Affluence Scale III [24]. The sum score of the six items showed high test–retest reliability (*r* = 0.90) and consistency between child and parent report (*r* = 0.80)
School pressure	“How pressured do you feel by the schoolwork you have to do?”	Not at all or a little (1), some (2), a lot (3)		
Physical activity	“Over the past 7 days, on how many days were you physically active for a total of at least 60 min per day?”	None (0) to seven (7) days		Biddle et al. [25] report a reasonable validity and moderate reliability for this index. Prochaska et al. [26] validated this measure for use with children and adolescents.
Screen time	“How many h a day, in your free time, do you usually spend watching television (TV), videos (including YouTube or similar services), DVDs, and other entertainment on a screen?”; “How many h a day, in your free time, do you usually spend playing games on a computer, games console, tablet (like iPad), smartphone or other electronic device (not including moving or fitness games)?”; and “How many h a day, in your free time, do you usually spend using electronic devices such as computers, tablets (like iPad) or smart phones for other purposes, for example, homework, emailing, tweeting, Facebook, chatting, surfing the Internet?”	None at all (1), “half an hour a day” (2), “1 h a day” (3), “2 h a day” (4), “3 h a day” (5), “4 h a day” (6), “5 h a day” (7), “6 h a day” (8), “7 h or more a day” (9)	Assessed separately for week- and weekend days. Response categories were transformed to number of h using the midpoint method. To represent the overall amount of screen time per day, the three screen time behaviours were summed for week- and weekend days separately. Finally, the mean screen time on weekdays and weekends together was calculated ((5 × weekdays) + (2 × weekend days)/7).	Rey-López JP et al (2010) [27]
Psychological symptoms	“In the last 6 months: how often have you had the following… (1) feeling low, (2) irritability or bad temper, and (3) feeling nervous?”	Five: “about every day” (1), “more than once a week” (2), “about every week” (3), “about every month” (4), and “rarely or never” (5)	Response categories were reversely scored to facilitate interpretation of the results.	Three psychological symptoms of the Health Behaviour in School-Aged Children (HBSC) Symptom Check List (HBSC-SCL) were used [28]. The HBSC-SCL consists of four questions on somatic and four questions on psychological symptom load. The internal validity of the psychological subscale was good (α = 0.78) [28]. When leaving out the symptom of sleep onset difficulties from the psychological symptom load generated by the HBSC-SCL, the internal consistency of the psychological dimension slightly improved (Cronbach’s alpha increased from 0.78 to 0.79).
Sleep onset difficulties	“In the last 6 months: how often have you had the following… difficulties in getting to sleep?”	Five: “about every day” (1), “more than once a week” (2), “about every week” (3), “about every month” (4), and “rarely or never” (5)	Response categories were reversely scored to facilitate interpretation of the results.	This is the fourth psychological symptom of the HBSC-SCL [28].
Sleep duration week	“When do you usually go to bed if you have to go to school the next morning?”	Eleven: ranging from “no later than 21.00” to “02.00 or later”	Response categories were transformed to h using the midpoint method.	Wolfson et al. found that school and weekend nights survey variables were significantly correlated with actigraphy and diary variables [29].
	“When do you usually wake up on school mornings?”	Seven: ranging from no later than 05.00” to “08.00 or later”	Sleep duration was calculated by subtracting the reported bedtime from the wake-up time.	
Sleep duration weekend	“When do you usually go to bed at weekends or during holidays?”	Fifteen: ranging from “no later than 21.00” to “04.00 or later”		
	“When do you usually wake up at weekends or during holidays?”	Fifteen: ranging from “no later than 05.00” to “14.00 or later”		

**Table 2:** Results of the mediation analysis.

		Sleep Duration on Weekdays	Sleep Duration on Weekend Days	Sleep Onset Difficulties
**Mediator model ^1^**		*β* (95% CI)	*β* (95% CI)	*β* (95% CI)
School pressure (ref. none or a little)	Some	−0.09 (−0.11; −0.07) *	−0.08 (−0.11; −0.04) *	0.32 (0.30; 0.35) *
	A lot	−0,25 (−0.28; −0.22) *	-0.20 (-0.24; -0.15) *	0.73 (0.69; 0.77) *
Physical activity		−0.01 (−0.01; −0.01) *	−0.03 (−0.04; −0.02) *	−0.03 (−0.03; −0.02) *
Screen time		−0.07 (−0.07; −0.07) *	−0.05 (−0.05; −0.05) *	0.04 (0.04; 0.05) *
**Outcome model ^2^**		−0.13 (−0.13; −0.12) *	−0.035 (−0.04; −0.03) *	0.30 (0.30; −0.31) *
**Mediating relationship**				
School pressure (ref. none or a little)	Some	0.01 (0.01; 0.01) *	0.003 (0.001; 0.004) *	0.10 (0.09; 0.11) *
	A lot	0.03 (0.03; 0.04) *	0.007 (0.005; 0.009) *	0.22 (0.21; 0.23) *
Physical activity		0.001 (0.001; 0.002) *	0.001 (0.001; 0.001) *	−0.01 (−0.01; −0.01) *
Screen time		0.009 (0.008; 0.009) *	0.002 (0.001; 0.002) *	0.01 (0.01; 0.01) *
**Direct relationship ^3^**				
School pressure (ref. none or a little)	Some	0.39 (0.37; 0.41) *	0.40 (0.38; 0.42) *	0.30 (0.28; 0.32) *
	A lot	0.83 (0.80; 0.85) *	0.85 (0.83; 0.89) *	0.64 (0.61; 0.66) *
Physical activity		−0.03 (−0.03; −0.02) *	−0.03 (−0.03; −0.02) *	−0.02 (−0.02; −0.01) *
Screen time		0.03 (0.03; 0.03) *	0.04 (0.04; 0.04) *	0.03 (0.02; 0.03) *
**% mediated**		%	%	%
School pressure (ref. none or a little)	Some	2.85 (2.21; 3.56) *	0.66 (0.37; 1.02) *	24.60 (22.28; 26.58) *
	A lot	3.62 (3.15; 4.08) *	0.79 (0.60; 1.03) *	25.80 (24.60; 27.20) *
Physical activity		Inconsistent mediation	Inconsistent mediation	33.56 (26.36; 41.61) *
Screen time		22.16 (20.29; 24.30) *	4.49 (3.73; 5.37) *	34.13 (31.74; 36.63) *

^1^ In the mediator model, the relationship between the independent variables (school pressure, physical activity, and screen time) and the mediating variables (sleep duration on week- and weekend days and sleep onset difficulties) was estimated. ^2^ In the outcome model, the relationship between the mediating variables (sleep duration on week- and weekend days and sleep onset difficulties) and the outcome psychological symptoms was estimated. ^3^ The direct relationship represents the relationship between the predictors school pressure, physical activity, screen time, and the outcome psychological symptoms that is not mediated through sleep duration on week- or weekend days or sleep onset difficulties. * All *p*-values were <0.001.
